# The role of neuronal activity and transmitter release on synapse formation^☆^

**DOI:** 10.1016/j.conb.2014.02.008

**Published:** 2014-08

**Authors:** Laura C Andreae, Juan Burrone

**Affiliations:** MRC Centre for Developmental Neurobiology, King's College London, New Hunt's House, 4th Floor, Guy's Hospital Campus, London SE1 1UL, UK

## Abstract

The long history of probing the role of neuronal activity in the development of nervous system circuitry has recently taken an interesting turn. Although undoubtedly activity plays a critical part in the maintenance and refinement of synaptic connections, often via competitive mechanisms, evidence is building that it also drives the process of synapse formation itself. Perhaps predictably, this turns out not to be a uniform process. It seems that different circuits, indeed specific synaptic connections, are differentially sensitive to the effects of activity. We examine possible ways in which neurotransmitter may drive synapse formation, and speculate on how the environment of the developing brain may allow a different spatiotemporal range for neuronal activity to operate in the generation of connectivity.

## Highlights

• Synaptic activity drives the formation of specific synapses in the retina.
• Neurotransmitter induces the formation of spines in developing cortical neurons.
• Axons are capable of releasing neurotransmitter before synaptic contacts.
• We speculate on the role of early, non-synaptic release in synaptogenesis.

**Current Opinion in Neurobiology** 2014, **27**:47–52

This review comes from a themed issue on **Development and regeneration**

Edited by **Oscar O Marín** and **Frank F Bradke**

For a complete overview see the Issue and the Editorial

Available online 13th March 2014

0959-4388/$ – see front matter, © 2014 The Authors. Published by Elsevier Ltd. All rights reserved.

**http://dx.doi.org/10.1016/j.conb.2014.02.008**

## Introduction

Ever since the classic experiments by Hubel and Wiesel where they demonstrated that visual experience is required to drive the formation of ocular dominance columns in the visual cortex [1,2], the role of neuronal activity in shaping developing circuits has been a subject of intense investigation. Over the years, it has become clear that in many developing systems, from the neuromuscular junction to the visual system, activity plays a key role in the refinement and maintenance of neuronal circuits, often via competitive mechanisms [3–5]. However, it is increasingly evident that neuronal activity, whether spontaneous or driven by sensory experience, is also important for the formation of synapses. In this review, we will focus on the role of activity in synapse formation, recent work on how these effects can be highly cell-type specific, and discuss possible mechanisms that link neurotransmitter release to the process of synaptogenesis.

## Activity regulates synapse formation

While it seems that synaptogenesis *per se* is able to proceed in the absence of neuronal activity [6,7], many studies that have interfered with activity have demonstrated a negative effect on synapse number. Genetic deletion of the Munc18-1 protein, which is critically required for correct assembly of the presynaptic release machinery, leads to complete abolition of neurotransmitter release [7]. Although morphologically normal synapses do develop in this mutant [7], there is a dramatic reduction in synapse number in the cortex [8]. Similarly, mice lacking ChAT, the biosynthetic enzyme for the neurotransmitter acetyl choline (ACh), lose all transmission at the neuromuscular junction and display smaller motor nerve terminals which make fewer synapses [9]. Many other tactics have been employed to interfere with neuronal transmission and the firing of action potentials, including more recently the incorporation of traditional neurotoxins or naturally occurring ion channels that hyperpolarise cells into genetically controlled expression systems to allow spatiotemporal control of neuronal/synaptic activity. For example, expression of the inwardly rectifying potassium channel Kir2.1 results in hyperpolarisation of neurons and hence reduced firing. When this was expressed in hippocampal neurons in culture, fewer synapses were formed on to the silenced cell [10]. However, these effects were likely to be competitive in nature as global silencing of action potential firing with tetrodotoxin, a blocker of sodium channels, rescued these changes. Tetanus toxin prevents neurotransmitter release by cleaving the presynaptic vesicle SNARE protein VAMP2. In the olfactory system, conditional silencing of olfactory sensory neurons (OSNs) was achieved using a cell-type specific promoter to drive either tetanus toxin light chain to prevent neurotransmitter release or Kir2.1 to electrically silence neurons [11]. Interestingly, while blockade of transmitter release had only competitive effects on postsynaptic targeting, global silencing with Kir2.1 led to a delay in axon entry and disorganised OSN targets, the glomeruli [11]. These studies emphasise the notion that differential effects of activity are seen in different contexts and using alternative methods of manipulating activity [12].

**Figure 1:**
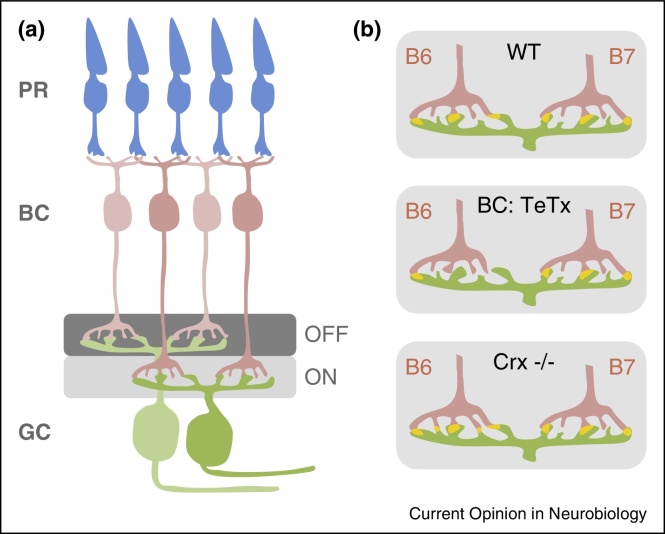
Synaptic activity drives the formation of synaptic contacts in the developing retina. **(a)** Schematic diagram of the retina showing photoreceptors (blue), bipolar cells (pink) and ganglion cells (green). Synapses between bipolar and ganglion cells occur in different layers, broadly divided into ON and OFF layers. **(b)** Synaptic contacts (yellow) formed between two different types of ON bipolar cells (B6 and B7, pink) and a specific type of ON ganglion cell (G10, green) are modulated by activity. Expressing tetanus toxin in B6 or B7 bipolar cells (middle panel) results in a specific decrease in the number of connections formed by B6 but not by B7 neurons. Conversely, increasing the activity of bipolar cells (bottom panel) causes an increase in the number of B6 synapses, but not those formed by B7 neurons.

A reduction in synapse number could be due to impaired synapse formation, but could alternatively be a result of increased rates of synapse elimination or a failure of maintenance. Almost 20 years ago, a study in ferrets mapped the location of a specific subset of synapes in developing and mature visual cortex and found evidence which suggested that activity could itself drive synaptogenesis [13]. With technical advances in imaging it has become possible to distinguish between these possibilities by employing careful timelapse imaging of neurons during the critical period of synaptogenesis. An early study examined turnover of the post-synaptic scaffolding protein, PSD-95, and found that blockade of activity led to a reduction in turnover [14] with a specific effect of antagonising NMDA receptors on inhibition of new cluster generation. More recently, this issue has been carefully addressed in the mouse retina. This system allows elegant genetic control of specific cell types, including presynaptic and postsynaptic partners, coupled with well described anatomical and functional connections. Furthermore, it has long been used to examine activity-dependent effects on circuits in general. In the retina, photoreceptors (rod or cone) connect to bipolar cells which in turn form excitatory synapses onto retinal ganglion cells (RGCs) (Figure 1a). A key study from the Wong laboratory silenced ON bipolar cells by expressing tetanus toxin under the mGluR6 promoter (*Grm6*-TeNT) [15]. These ON bipolar cells normally project to mono-stratified ON RGCs or to part of the dendritic tree of bi-stratified ON/OFF RGCs. Although the dendrites of the ON RGCs showed normal stratification, there was a 50% reduction in synapse number from ON bipolar cells. Interestingly, bi-stratified ON/OFF RGCs showed a reduction in ON synapses with no effect on OFF connections, suggesting that these two populations of synaptic connections seem to form independently. Live imaging at P9 demonstrated a significant reduction in the rate of synapse formation, with normal levels of elimination, indicating that excitatory synaptic activity from the ON bipolar cell regulates synapse number onto its target specifically via effects on synapse formation.

## Cell-type specific pathways

Taking advantage of the ability to fluorescently label specific subtypes of cells and their connections in the mouse retina, a number of recent studies have demonstrated that the effects of activity are exerted in a highly differential manner [16^••^,17^•^,18^•^]. Detailed mapping of normal developmental changes indicated that the B6 bipolar cell nearly doubles the number of connections with G10 RGCs between P9 and P21, while the B7 bipolar cell maintains a relatively constant number and the RB bipolar cell loses its connections. Silencing bipolar cells with tetanus toxin had completely different effects on each type of bipolar cell: a dramatic reduction in B6-G10 synapses at P21, but no effect on B7-G10 synapses (Figure 1b) and the RB-G10 synapses were correctly eliminated [16^••^]. Similarly, another study examined the effects of sensory deprivation (dark rearing) on the photoreceptor-to-bipolar cell connection, this time looking at rod versus cone photoreceptors synapsing on to B6,7 or 8 bipolar cells. Once again, they found differential (and complex) effects of sensory deprivation on different connections [18^•^], which interestingly is reflected in different spatiotemporal patterns of dendritic arbor growth [19^•^].

Most studies examining activity-dependent effects have used different techniques to silence activity, but a recent study set out to determine whether increasing activity could lead to a corresponding increase in synapse formation [17^•^]. The Crx^−/−^ mutant mouse displays increased spontaneous firing of RGCs after eye opening (∼P15), due to increased glutamate release from bipolar cells. From this stage onwards, mutants show increasing numbers of bipolar-to-RGC synapses. Live imaging during this period indicates that this increase is due to higher rates of synapse formation, with no impact on elimination rates. Further, there were dramatically different effects in each cell type: B6-G10 synapses showed a doubling in synapse number, while there was no effect on B7/RB-G10 synapses (Figure 1b) [17^•^].

The finding that activity differentially affects distinct cell types, even when examining different axonal inputs onto the same postsynaptic target, may go some way to explaining why activity blockade spares many synaptic connections. But why, for example, does activity regulate B6 connections onto G10 RGCs, but not those of B7 and RB cells? Activity levels seem also to differentially affect excitatory and inhibitory synapses. In the adult mouse, depriving one eye of vision has recently been found to lead to a reduction in inhibitory synapse formation [20] (and increased rates of loss [21]) whereas excitatory synapses increase [22]. This is perhaps not surprising: excitatory and inhibitory synapses not only release different neurotransmitters with (usually) opposing effects on the postsynaptic cell, but also express different proteins with differing sensitivities to manipulation (e.g. [6]) and show different behaviours during early synapse formation [23]. Clearly, different circuits subserve different roles, even where they converge on the same cells, and there are likely to be key developmental and functional reasons why some need to be regulated by activity and some do not. How and why this occurs will be an area for much future investigation.

## What mechanisms could be responsible for activity-driven synapse formation?

Before synapses are formed, axonal growth cones have long been known to release neurotransmitter [24,25], even before synaptic vesicle proteins are expressed [26]. Experiments in Xenopus embryonic spinal cord neurons showed that both evoked and spontaneous neurotransmitter release occurred all along the axons of isolated neurons, including growth cones [25,27]. Indeed, high levels of on-going vesicle cycling have also been shown to exist in young axons and growth cones. In dissociated hippocampal neurons, constitutive vesicle cycling was observed in growing axons [28], as well as in the long thin filopodia of growth cones [29]. More recent work has shown that young axons preferentially undergo spontaneous forms of vesicle cycling, gradually switching towards higher levels of evoked release during development. This switch in vesicle cycling modes was cell autonomous and independent of postsynaptic contact [30^•^]. Although most studies into the role of activity on synapse and circuit development have focused on action potential firing, a few studies have demonstrated effects of spontaneous release during development, on dendritic spine maintenance [31] and on modulating the dendritic arbor response to the neurotrophin BDNF [32]. Given the high levels of spontaneous, quantal release in developing systems [30^•^,33] it will be of interest to see if differential roles can be identified for different types of presynaptic transmitter release.

At this early stage in development, dendrites are studded with highly motile protrusions, or filopodia [34]. These filopodia are known to be precursors to dendritic spines [35]. Immature dendrites are also known to express transmitter receptors prior to innervation, such as the NMDA glutamate receptor [36], which undergo constant cycling with the plasma membrane even at these early stages [37]. It is tempting to imagine that the early release of neurotransmitters could exert some kind of effect on these filopodia that might regulate synaptogenesis. Activation of NMDA receptors early in development, in some cases prior to synapse formation, has been observed in different preparations [38–40]. Further, glutamate itself can induce filopodia formation [41] and increase filopodial length [42], while blockade of NMDA receptors or metabotropic glutamate receptors has the reverse effect [42,43]. In the chick retina, glutamate receptor blockade at a specific timepoint in development impaired filopodial extension rates and amounts (although retraction was also affected) [44]. *In vivo* studies in Xenopus have shown that visual stimulation can increase dendritic branch formation, potentially either a precursor of or a sequel to synapse formation [45,46], which is prevented by antagonising both NMDA and AMPA glutamate receptors [47]. In fact, impairment of filopodial motility (due to deletion of the protein EphB2) is associated with a dramatic reduction in synapse density due to impaired synaptogenesis [48]. Thus it seems that presynaptic activity could operate on dendritic receptors, particularly those of the NMDA subtype, to regulate filopodia and hence have effects on dendritic branching and presumably, synapse formation.

**Figure 2:**
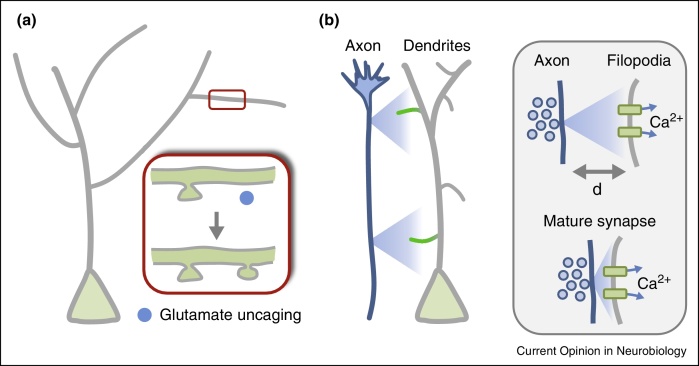
The role of the neurotransmitter glutamate in synapse formation. **(a)** Glutamate can induce the formation of functional postsynaptic spines. The diagram shows a developing neuron in the cortex with a cell body (green) and apical dendrites (gray). The inset shows a zoomed in section of the dendrite (red box), which contains an existing spine (top drawing). Glutamate is uncaged locally with a laser (blue circle), resulting in the emergence of a new postsynaptic spine. Adapted from [53]. **(b)** Long range communication between neurons in developing circuits. Growing axons release neurotransmitter before they form any synaptic connections (indicated by graded blue signal from axons). Filopodia from neighbouring neurons may be able to sense this neurotransmitter at a distance (indicated by the graded green response in filopodia). Inset: the release of neurotransmitter, such as glutamate, from presynaptic vesicles (blue) clustered along a growing axon could activate distant dendritic receptors (green), such as NMDA receptors, resulting in possible calcium influx and plasticity (top drawing). Such a form of long-range communication, distinct from local synaptic transmission in mature synapses (bottom drawing) could provide information for circuit assembly and synapse formation. The spatial extent of this form of communication (d) may well be larger than the few nanometers of mature synapses and may help instruct postsynaptic filopodia and dendrites during the process of synaptogenesis.

Studies from the field of synaptic plasticity have revealed that high levels of activity (able to induce long-term potentiation of synaptic strength) can induce new spine formation [49] and indeed NMDA-dependent dendritic filopodial growth [50]. It seems intuitive that Hebbian-like plasticity mechanisms could play a role during earlier synapse development [51,52]. A more recent study has now shown that neurotransmitter can directly induce the *de novo* formation of mature spines during development in cortical neurons (Figure 2a) [53^••^]. Either focal uncaging of caged glutamate close to a stretch of dendrite (less than 1 μm away) or high frequency stimulation resulted in the local growth of spines very rapidly (within 6 s of the uncaging protocol). This effect was dependent on NMDA receptor activation. Surprisingly, the new spines did not emerge as filopodia, but as mature spines, both structurally and functionally, expressing receptors and channels that allows their rapid integration into the circuit. How these experiments compare to the earlier stages of synapse formation where immature axons have not yet established any synaptic contact, is not yet clear. Also, whether neurotransmitter release could act at a distance, and how far a release site needs to be to activate postsynaptic receptors, remains unknown. Future studies are likely to illuminate further possible mechanisms for activity-driven synaptogenesis.

## How early can neurotransmitter release influence synapse formation?

The fact that axons release neurotransmitter even before synapsing onto their target dendrites raises the possibility that the process of activity-dependent synapse formation may actually begin very early on, before contacts are made. For this to occur, axons may well relay information at a distance, from a growing axon to a nearby dendrite or filopodium and influence its activity in a meaningful manner (Figure 2b). However, the distance over which neurotransmitter can influence neighbouring neurons during development is unknown. Although transmission is highly localised in mature synapses there are many reasons to believe that this may not be the case early in circuit development. For a start, the expression of transporters that would normally curtail the spread of neurotransmitter from its source is delayed until after synapses have begun to form, increasing the area of influence from a single release site [54,55]. Additionally, developing neurons preferentially express NMDARs containing the NR2B subunit, which has a higher sensitivity to both glutamate and its co-agonist glycine when compared to NR2A-containing receptors that are mainly found in mature synaptic contacts, making developing dendrites highly sensitive to glutamate [56]. Critically, many studies have found that blockade of glutamate transporters during development can induce dramatic synchronous oscillations of neurons which are dependent on NMDA receptor activation [57–59], indicating that extrasynaptic or ‘spillover’ glutamate is present in the developing brain. Indeed, a series of recent studies have compellingly demonstrated the presence of activity-dependent glutamate spillover using outside-out patches or optical sensors, acting via NMDA receptors in developing retinae, which was important for correct spatiotemporal retinal wave structure [60,61^•^], as well as extracellular ACh associated with cholinergic waves [62^•^]. Furthermore, there is evidence showing that the neurotransmitters GABA and glutamate can be released in a paracrine fashion and act on postsynaptic receptors in developing systems [63]. Interestingly, this study found that even in neurons prior to synapse formation, ambient GABA (but not glutamate) had tonic activation effects on membrane potentials [63]. GABA is well known to exert multiple trophic actions on neuronal development, including increasing synapse density [64]. Together, these findings suggest that the release of neurotransmitter from a growing axon could, in principle, act at a distance to influence dendritic growth. Such a mechanism could have an important impact on how we think about the spatial domain over which synaptic transmission can operate (long-range, versus direct contact) and the temporal domain (early in development) over which neurotransmitter release can influence dendritic structures.

Studies investigating the role of neuronal activity in the wiring of the brain have mainly focused on the process of circuit refinement that ensues after synapses have formed. More recent findings have shifted the focus of attention to earlier events that occur concurrently with, or immediately preceding, synapse formation and influence the actual process of synaptogenesis itself. The wealth of new molecular and imaging tools to visualise the process of synapse formation will undoubtedly shed light on the critical periods needed for activity-dependent synapse formation and help us understand how and when neurons communicate with each other to form functional synaptic contacts.

## References and recommended reading

Papers of particular interest, published within the period of review, have been highlighted as:

• of special interest
•• of outstanding interest
